# PMab-219: A monoclonal antibody for the immunohistochemical analysis of horse podoplanin

**DOI:** 10.1016/j.bbrep.2019.01.009

**Published:** 2019-02-02

**Authors:** Yoshikazu Furusawa, Shinji Yamada, Shunsuke Itai, Takuro Nakamura, Miyuki Yanaka, Masato Sano, Hiroyuki Harada, Masato Fukui, Mika K. Kaneko, Yukinari Kato

**Affiliations:** a Department of Antibody Drug Development, Tohoku University Graduate School of Medicine, 2-1 Seiryo-machi, Aoba-ku, Sendai, Miyagi, 980-8575, Japan; b New Industry Creation Hatchery Center, Tohoku University, 2-1 Seiryo-machi, Aoba-ku, Sendai, Miyagi, 980-8575, Japan; c ZENOAQ RESOURCE CO., LTD, 1-1 Tairanoue, Sasagawa, Asaka-machi, Koriyama, Fukushima, 963-0196, Japan; d Department of Oral and Maxillofacial Surgery, Graduate School of Medical and Dental Sciences, Tokyo Medical and Dental University, 1-5-45, Yushima, Bunkyo-ku, Tokyo, 113-8510, Japan

**Keywords:** Horse podoplanin, PDPN, PMab-219, CBIS, Cell-Based Immunization and Screening, CHO, Chinese hamster ovary, CLEC-2, C-type lectin-like receptor-2, DAB, 3,3'-diaminobenzidine tetrahydrochloride, ELISA, enzyme-linked immunosorbent assay, horPDPN, horse podoplanin, hPDPN, human podoplanin, mAb, monoclonal antibody, PBS, phosphate-buffered saline, PDPN, podoplanin, PVDF, polyvinylidene difluoride, SDS, sodium dodecyl sulfate

## Abstract

Monoclonal antibodies (mAbs) against human, mouse, rat, rabbit, dog, cat, and bovine podoplanin (PDPN), a lymphatic endothelial cell marker, have been established in our previous studies. However, mAbs against horse PDPN (horPDPN), which are useful for immunohistochemical analysis, remain to be developed. In the present study, mice were immunized with horPDPN-overexpressing Chinese hamster ovary (CHO)-K1 cells (CHO/horPDPN), and hybridomas producing mAbs against horPDPN were screened using flow cytometry. One of the mAbs, PMab-219 (IgG_2a_, kappa), specifically detected CHO/horPDPN cells via flow cytometry and recognized horPDPN protein using Western blotting. Furthermore, PMab-219 strongly stained CHO/horPDPN via immunohistochemistry. These findings suggest that PMab-219 is useful for investigating the function of horPDPN.

## Highlights

• PDPN is known as a specific lymphatic endothelial cell (LEC) marker.
• Sensitive and specific PMab-219 mAb against horse PDPN was produced.
• PMab-219 reacted with a horse renal cell line sensitively in flow cytometry.
• PMab-219 is useful for IHC using paraffin-embedded cell sections.

## 1 Introduction

Podoplanin (PDPN), a type I transmembrane glycoprotein, is expressed in normal tissues including renal podocytes, type I lung alveolar cells, and lymphatic endothelial cells [1,2]. The interaction between PDPN on lymphatic endothelial cells and C-type lectin-like receptor-2 (CLEC-2) on platelets has been shown to facilitate embryonic blood/lymphatic vessel separation [1,[3], [4], [5], [6], [7], [8], [9], [10]].

The expression of human PDPN (hPDPN) has been reported in several malignant tumors, including oral squamous cell carcinomas [11], esophageal cancers [12], lung cancers [13], malignant mesotheliomas [14,15], osteosarcomas [[16], [17], [18]], chondrosarcomas [17], malignant brain tumors [[19], [20], [21], [22]], and testicular tumors [23]. The expression of hPDPN is associated with malignant progression and cancer metastasis [6,19,24].

Until now, we have developed monoclonal antibodies (mAbs) against human [25], mouse [25], rat [26], rabbit [27], bovine [28], dog [29], and cat [30] PDPNs. Furthermore, an anti-cat PDPN mAb (PMab-52) cross-reacted with tiger PDPN [31]. Although an anti-horse PDPN (horPDPN) mAb, PMab-202 was recently established by immunizing mice with synthetic peptides of horPDPN, it was not useful for immunohistochemical analysis [32]. Sensitive and specific mAbs against horPDPN are necessary to investigate the expression and function of horPDPN. In the present study, we immunized mice with CHO/horPDPN cells and established hybridomas that could produce mAbs against horPDPN.

## 2 Materials and methods

### 2.1 Cell lines

CHO-K1 and P3X63Ag8U.1 (P3U1) cells were obtained from the American Type Culture Collection (ATCC, Manassas, VA, USA). The horse kidney cell line, FHK-Tcl3.1, was established at Yamaguchi University [33]. The horPDPN bearing an N-terminal PA16 tag (PA16-horPDPN) was inserted into a pCAG-Ble vector (FUJIFILM Wako Pure Chemical Corporation, Osaka, Japan) [32]. The PA16 tag comprises 16 amino acids (GLEGGVAMPGAEDDVV) [34]. CHO-K1 cells were transfected with pCAG-Ble/PA16-horPDPN using Lipofectamine LTX with Plus Reagent (Thermo Fisher Scientific Inc., Waltham, MA, USA). Stable transfectants were selected by limiting dilution and cultivated in a medium containing 0.5 mg/mL of zeocin (InvivoGen, San Diego, CA, USA).

P3U1, CHO-K1, and CHO/horPDPN cells were cultured in Roswell Park Memorial Institute (RPMI) 1640 medium (Nacalai Tesque, Inc., Kyoto, Japan), and FHK-Tcl3.1 was cultured in Dulbecco's modified Eagle's medium (DMEM; Nacalai Tesque, Inc.) [32]. All media were supplemented with 10% heat-inactivated fetal bovine serum (Thermo Fisher Scientific Inc.), 100 units/mL of penicillin, 100 μg/mL of streptomycin, and 25 μg/mL of amphotericin B (Nacalai Tesque, Inc.). Cells were grown at 37 °C in a humidified environment with an atmosphere of 5% CO_2_ and 95% ambient air.

### 2.2 Animals

Female BALB/c mice (6 weeks old) were purchased from CLEA Japan (Tokyo, Japan). Animals were housed under specific pathogen-free conditions. The Animal Care and Use Committee of Tohoku University approved all the animal experiments.

### 2.3 Hybridoma production

Two BALB/c mice were immunized with CHO/horPDPN cells (1 × 10^8^), which were intraperitoneally (i.p.) administered together with Imject Alum (Thermo Fisher Scientific Inc.). The procedure included an additional three immunizations followed by a final booster injection administered i.p. 2 days prior to the harvest of spleen cells, making a total of five immunizations. Subsequently, these spleen cells were fused with P3U1 cells using PEG1500 (Roche Diagnostics, Indianapolis, IN, USA), and the hybridomas were grown in RPMI medium supplemented with hypoxanthine, aminopterin, and thymidine for selection (Thermo Fisher Scientific Inc.). The culture supernatants were screened using flow cytometry.

### 2.4 Flow cytometry

The cells were harvested following brief exposure to 0.25% trypsin/1 mM EDTA (Nacalai Tesque, Inc.). The cells were washed with 0.1% BSA/PBS and treated with primary mAbs for 30 min at 4 °C. Thereafter, the cells were treated with Alexa Fluor 488-conjugated anti-mouse IgG (1:2000; Cell Signaling Technology, Inc., Danvers, MA, USA) or Oregon green anti-rat IgG (1:2000; Thermo Fisher Scientific Inc.). Fluorescence data were collected using SA3800 Cell Analyzers (Sony Corp., Tokyo, Japan).

### 2.5 Determination of binding affinity using flow cytometry

CHO/horPDPN or FHK-Tcl3.1 (2 × 10^5^ cells) was suspended in 100 μL of serially diluted PMab-219, followed by addition of Alexa Fluor 488-conjugated anti-mouse IgG (1:200; Cell Signaling Technology, Inc.). Fluorescence data were collected using EC800 Cell Analyzer (Sony Corp.). The dissociation constant (*K*_D_) was obtained by fitting the binding isotherms to built-in one-site binding models in GraphPad PRISM 6 (GraphPad Software, Inc., La Jolla, CA, USA).

### 2.6 Western blotting

Cell lysates (10 μg) were boiled in sodium dodecyl sulfate (SDS) sample buffer (Nacalai Tesque, Inc.). The proteins were subjected to electrophoresis on 5%–20% polyacrylamide gels (FUJIFILM Wako Pure Chemical Corporation) and subsequently transferred onto a polyvinylidene difluoride (PVDF) membrane (Merck KGaA, Darmstadt, Germany). After blocking with 4% skim milk (Nacalai Tesque, Inc.), each membrane was incubated with primary mAbs, such as 1 μg/mL of PMab-219, 1 μg/mL of anti-PA16 tag (NZ-1), or 1 μg/mL of anti-*β*-actin (AC-15; Sigma-Aldrich Corp., St. Louis, MO, USA), and subsequently with peroxidase-conjugated anti-mouse IgG (1:1000; Agilent Technologies, Santa Clara, CA, USA) or anti-rat IgG (1:10000; Sigma-Aldrich Corp.). Bands were visualized with ImmunoStar LD (FUJIFILM Wako Pure Chemical Corporation) using a Sayaca-Imager (DRC Co. Ltd., Tokyo, Japan).

### 2.7 Immunohistochemical analyses

Cell blocks were produced using iPGell (Genostaff Co., Ltd., Tokyo, Japan) and processed to make 4-μm paraffin-embedded cell sections that were directly autoclaved in citrate buffer (pH 6.0; Nichirei Biosciences, Inc., Tokyo, Japan) for 20 min. These tissue sections were blocked using SuperBlock T20 (PBS) Blocking Buffer (Thermo Fisher Scientific Inc.), incubated with PMab-219 (1 μg/mL) for 1 h at room temperature, and treated using an Envision + Kit (Agilent Technologies Inc.) for 30 min. Color was developed using 3,3′-diaminobenzidine tetrahydrochloride (Agilent Technologies Inc.) for 2 min, and counterstaining was performed using hematoxylin (FUJIFILM Wako Pure Chemical Corporation).

## 3 Results and discussion

**Fig. 1:**
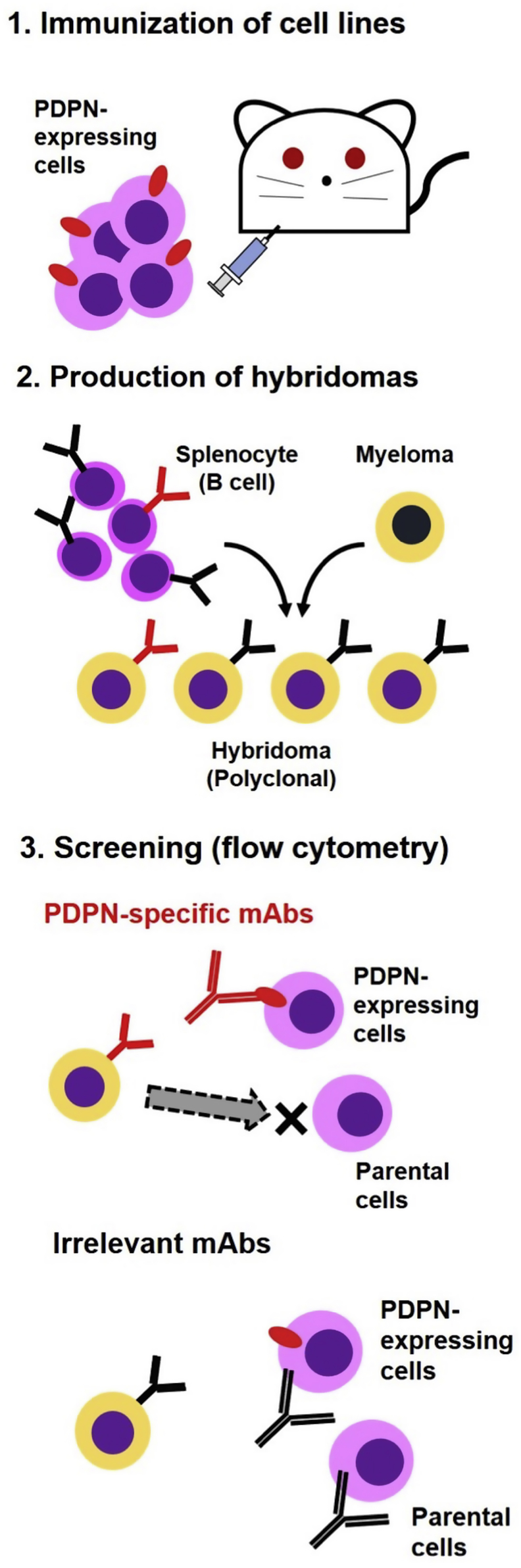
**Schematic illustration of Cell-Based Immunization and Screening (CBIS) method.** Stable transfectants expressing the protein of interest are used as an immunogen with no purification procedure. The selection of hybridomas secreting specific mAbs is performed by flow cytometry using parental and transfectant cells.

In the present study, we employed a Cell-Based Immunization and Screening (CBIS) method to develop sensitive and specific mAbs against horPDPN to facilitate the immunohistochemical analysis of paraffin-embedded tissue sections. Previously, we have successfully utilized a CBIS method to establish mAbs against various membrane proteins such as cat PDPN [30], CD44 [34], CD133 [35], and PD-L1 [36]. Two mice were immunized with CHO/horPDPN cells using an immunization and screening procedure (Fig. 1). Developed hybridomas were seeded into 96-well plates and cultivated for 10 days. Wells positive for CHO/horPDPN and negative for CHO-K1 were selected using flow cytometry. Moreover, FHK-Tcl3.1 cells were used to identify antibodies that reacted with the endogenous horPDPN. Screening identified strong signals against CHO/horPDPN cells and weak or no signals against CHO-K1 cells in 19 of 960 wells (2.0%). Of these 19 wells, nine hybridomas were developed: one clone of IgG_1_, one clone of IgG_2a_, five clones of IgG_3_, and two clones of IgM. One of these nine clones, PMab-219 (IgG_2a_, kappa), was finally selected using immunohistochemistry against horse tissues.

**Fig. 2:**
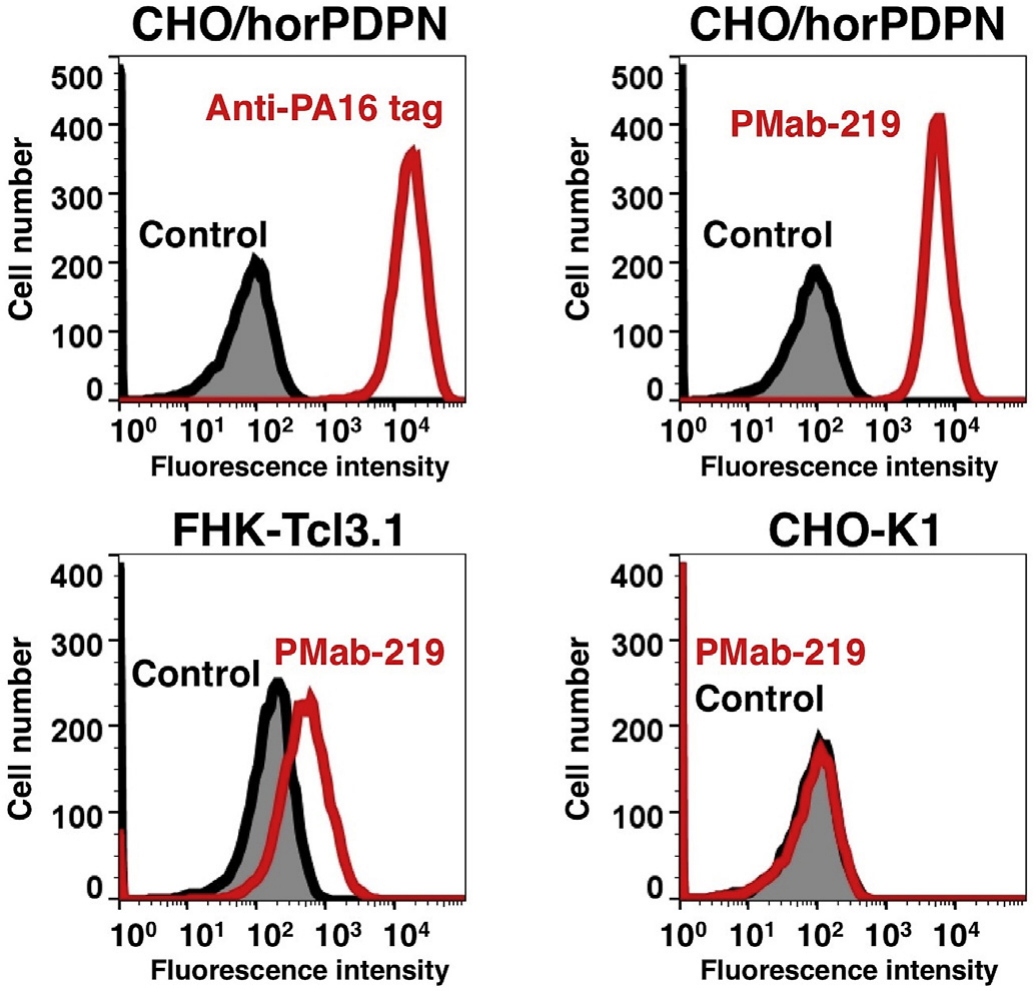
**Detection of horPDPN via flow cytometry using PMab-219.** CHO/horPDPN, CHO-K1, and FHK-Tcl3.1 cells were treated with PMab-219 (red line) or anti-PA16 tag (NZ-1; red line) at a concentration of 1 μg/mL or 0.1% BSA in PBS (gray) for 30 min, followed by incubation with secondary antibodies. (For interpretation of the references to colour in this figure legend, the reader is referred to the Web version of this article.)

PMab-219 recognized CHO/horPDPN but showed no reaction with CHO-K1, as assessed using flow cytometry (Fig. 2). PMab-219 reacted with FHK-Tcl3.1 cells, indicating that PMab-219 was able to recognize endogenous horPDPN. PMab-219 did not react with human, mouse, rat, rabbit, dog, cat, or bovine PDPNs (data not shown). Furthermore, it did not react with pig, Tasmanian devil, tiger, alpaca, bear, goat, sheep, or whale PDPNs (data not shown), indicating that PMab-219 is specific to horPDPN.

Additionally, a kinetic analysis conducted using flow cytometry assessed the interaction of PMab-219 with CHO/horPDPN and FHK-Tcl3.1 cells. *K*_D_ of PMab-219 for CHO/horPDPN and FHK-Tcl3.1 cells were determined to be 8.6 × 10^−8^ and 6.1 × 10^−7^ M, respectively, indicating moderate and low affinity for CHO/horPDPN and FHK-Tcl3.1 cells, respectively.

**Fig. 3:**
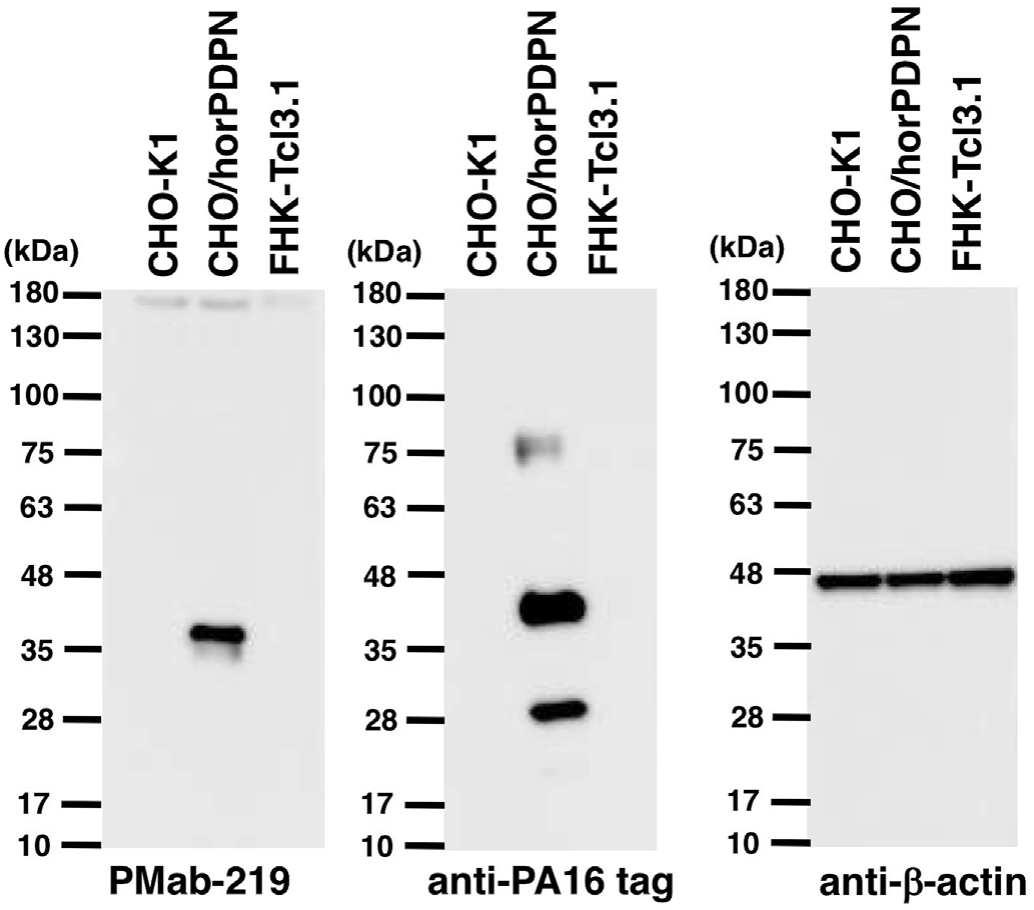
**Western blotting.** Cell lysates of CHO-K1, CHO/horPDPN, and FHK-Tcl3.1 (10 μg) were electrophoresed and transferred onto PVDF membranes. The membranes were incubated with l μg/mL of PMab-219, 1 μg/mL of anti-PA16 tag (NZ-1), or 1 μg/mL of anti-*β*-actin and subsequently with peroxidase-conjugated anti-mouse or -rat IgG.

Western blotting performed using PMab-219 (Fig. 3) demonstrated that PMab-219 detects horPDPN as a 40 kDa band in CHO/horPDPN cells. However, PMab-219 did not detect a 40 kDa band in FHK-Tcl3.1 cells; this might be attributed to low expression levels of horPDPN in FHK-Tcl3.1 cells. NZ-1, an anti-PA16 tag mAb, detected 40 and 25 kDa bands in CHO/horPDPN cells. The 40 kDa band represents a highly glycosylated form, whereas the 25 kDa band represents an unglycosylated one [3,22].

**Fig. 4:**
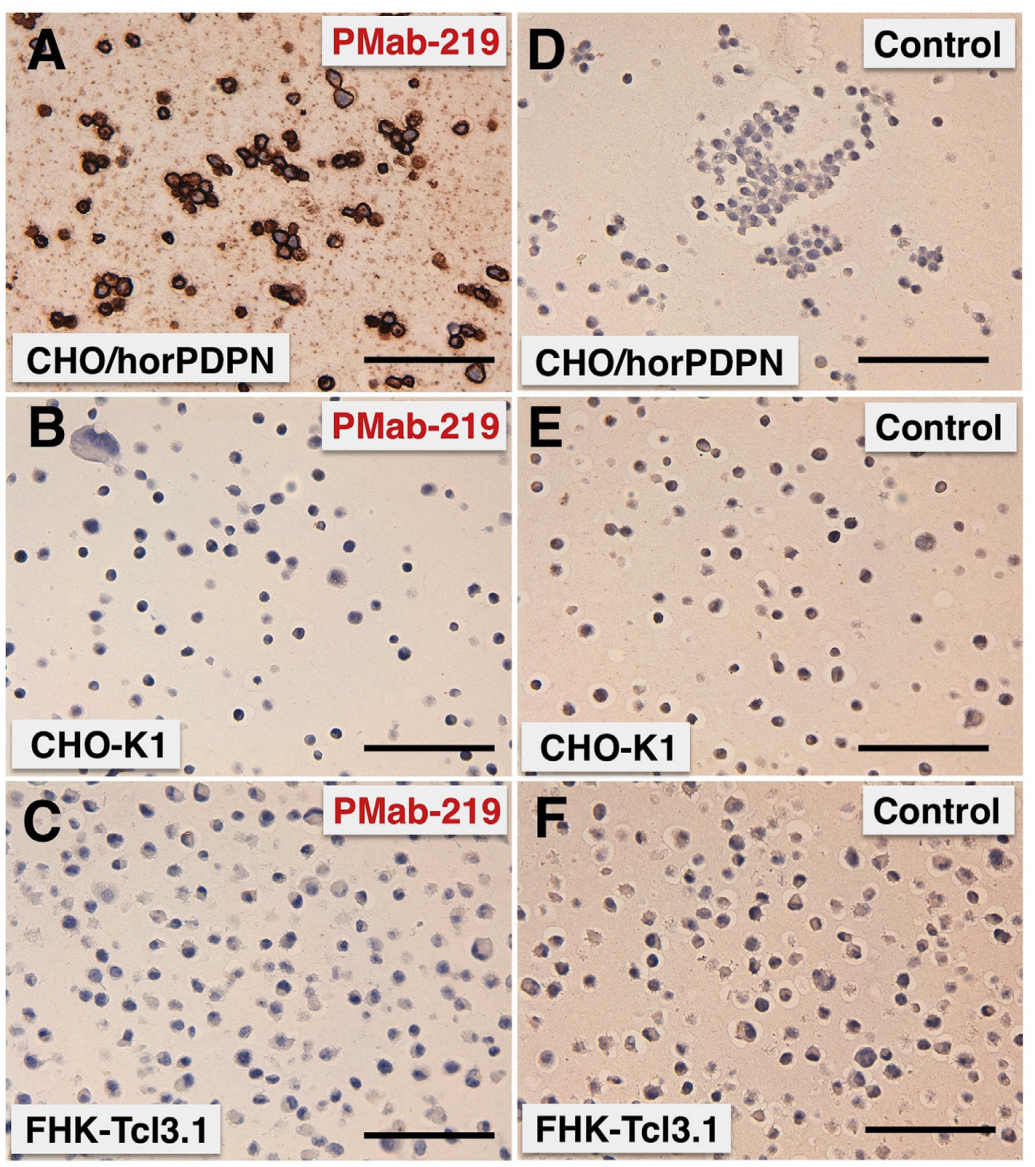
**Immunohistochemical analyses.** Cell sections of CHO/horPDPN (A, D), CHO-K1 (B, E), or FHK-Tcl3.1 (C, F) were incubated with 1 μg/mL of PMab-219 (A–C) or with blocking buffer (D–F), followed by an Envision + kit. Scale bar = 100 μm.

The immunohistochemical analyses revealed that PMab-219 strongly stained CHO/horPDPN cells (Fig. 4A) and did not react with CHO-K1 cells (Fig. 4B). PMab-219 did not react with FHK-Tcl3.1 cells; this might be also attributed to low expression levels of horPDPN in FHK-Tcl3.1 cells. (Fig. 4C). No staining was observed without primary antibodies (Fig. 4D, 4E, 4F). These results indicated that PMab-219 is useful for the detection of horPDPN using immunohistochemistry.

In conclusion, we have established an mAb against horPDPN, PMab-219, which is suitable for use in flow cytometry, Western blotting, and immunohistochemical analyses. The epitope of PMab-219 needs further investigation to clarify the sensitivity and specificity of PMab-219 against horPDPN. PMab-219 should prove useful for elucidating the pathophysiological functions of horPDPN in future studies.

## Funding

This research was supported in part by AMED under Grant Numbers: JP18am0101078 (Y.K.), JP18am0301010 (Y.K.), and JP18ae0101028 (Y.K.), and by JSPS KAKENHI Grant Number 17K07299 (M.K.K.) and Grant Number 16K10748 (Y.K.).

## Conflict of Interest

The authors declare no conflicts of interest involving this article.
